# Supplementary material for: Modeling glioblastoma heterogeneity as a dynamic network of cell states
Source: Mol Syst Biol. 2021 Sep 16;17(9):e10105. doi: 10.15252/msb.202010105 (PMC8444284; doi:10.15252/msb.202010105)
Supplement: Supplementary file 6 — Source Data for Figure 5 [file MSB-17-e10105-s004.zip › Figure5A_sourcedata/GSEA_3071/hallmarks_stateIV.GseaPreranked.1623742551702/HALLMARK_GLYCOLYSIS.html]

Details for gene set HALLMARK\_GLYCOLYSIS[GSEA]

|  || Dataset | state53071 |
| Phenotype | NoPhenotypeAvailable |
| Upregulated in class | na\_neg |
| GeneSet | HALLMARK\_GLYCOLYSIS |
| Enrichment Score (ES) | -0.37533385 |
| Normalized Enrichment Score (NES) | -1.973421 |
| Nominal p-value | 0.0 |
| FDR q-value | 0.0017807599 |
| FWER p-Value | 0.021 |
Table: GSEA Results Summary

  

Fig 1: Enrichment plot: HALLMARK\_GLYCOLYSIS      
 Profile of the Running ES Score & Positions of GeneSet Members on the Rank Ordered List

  

| PROBE | GENE SYMBOL | GENE\_TITLE | RANK IN GENE LIST | RANK METRIC SCORE | RUNNING ES | CORE ENRICHMENT || 1 | IGFBP3 |  |  | 59 | 1.794 | 0.0127 | No |
| 2 | CTH |  |  | 60 | 1.774 | 0.0365 | No |
| 3 | STC1 |  |  | 97 | 1.547 | 0.0503 | No |
| 4 | CXCR4 |  |  | 197 | 1.269 | 0.0483 | No |
| 5 | SOX9 |  |  | 214 | 1.223 | 0.0616 | No |
| 6 | B3GAT1 |  |  | 263 | 1.117 | 0.0674 | No |
| 7 | CAPN5 |  |  | 383 | 0.923 | 0.0569 | No |
| 8 | PAM |  |  | 431 | 0.871 | 0.0595 | No |
| 9 | IDH1 |  |  | 469 | 0.842 | 0.0637 | No |
| 10 | SDC2 |  |  | 571 | 0.747 | 0.0543 | No |
| 11 | CHPF |  |  | 621 | 0.713 | 0.0544 | No |
| 12 | EXT1 |  |  | 744 | 0.636 | 0.0394 | No |
| 13 | B3GAT3 |  |  | 856 | 0.577 | 0.0258 | No |
| 14 | MXI1 |  |  | 944 | 0.546 | 0.0164 | No |
| 15 | STC2 |  |  | 972 | 0.535 | 0.0184 | No |
| 16 | VEGFA |  |  | 1010 | 0.518 | 0.0182 | No |
| 17 | ZNF292 |  |  | 1153 | 0.479 | -0.0027 | No |
| 18 | IRS2 |  |  | 1218 | 0.458 | -0.0089 | No |
| 19 | CASP6 |  |  | 1265 | 0.450 | -0.0117 | No |
| 20 | GPC4 |  |  | 1269 | 0.448 | -0.0063 | No |
| 21 | HS2ST1 |  |  | 1273 | 0.446 | -0.0009 | No |
| 22 | HOMER1 |  |  | 1423 | 0.411 | -0.0240 | No |
| 23 | GNPDA1 |  |  | 1532 | 0.382 | -0.0397 | No |
| 24 | PPP2CB |  |  | 1580 | 0.370 | -0.0438 | No |
| 25 | AGL |  |  | 1699 | 0.349 | -0.0618 | No |
| 26 | AK3 |  |  | 1735 | 0.343 | -0.0639 | No |
| 27 | COG2 |  |  | 1856 | 0.322 | -0.0827 | No |
| 28 | VLDLR |  |  | 1866 | 0.319 | -0.0802 | No |
| 29 | SAP30 |  |  | 1914 | 0.311 | -0.0850 | No |
| 30 | GLCE |  |  | 2028 | 0.292 | -0.1029 | No |
| 31 | DPYSL4 |  |  | 2060 | 0.288 | -0.1050 | No |
| 32 | NOL3 |  |  | 2191 | 0.269 | -0.1264 | No |
| 33 | CD44 |  |  | 2197 | 0.268 | -0.1238 | No |
| 34 | CACNA1H |  |  | 2448 | -0.263 | -0.1684 | No |
| 35 | NANP |  |  | 2493 | -0.268 | -0.1732 | No |
| 36 | PGM2 |  |  | 2504 | -0.270 | -0.1715 | No |
| 37 | AKR1A1 |  |  | 2719 | -0.296 | -0.2088 | No |
| 38 | FUT8 |  |  | 2853 | -0.313 | -0.2302 | No |
| 39 | PSMC4 |  |  | 2891 | -0.318 | -0.2330 | No |
| 40 | PGK1 |  |  | 3257 | -0.369 | -0.2983 | No |
| 41 | P4HA1 |  |  | 3355 | -0.382 | -0.3119 | No |
| 42 | CYB5A |  |  | 3418 | -0.394 | -0.3185 | No |
| 43 | PLOD2 |  |  | 3453 | -0.400 | -0.3197 | No |
| 44 | SDC3 |  |  | 3547 | -0.419 | -0.3320 | No |
| 45 | HSPA5 |  |  | 3555 | -0.419 | -0.3277 | No |
| 46 | GMPPA |  |  | 3571 | -0.423 | -0.3249 | No |
| 47 | FKBP4 |  |  | 3632 | -0.433 | -0.3306 | No |
| 48 | RBCK1 |  |  | 3712 | -0.448 | -0.3398 | No |
| 49 | KIF20A |  |  | 3769 | -0.459 | -0.3445 | No |
| 50 | PPIA |  |  | 3778 | -0.461 | -0.3398 | No |
| 51 | MDH2 |  |  | 3798 | -0.466 | -0.3372 | No |
| 52 | TALDO1 |  |  | 3957 | -0.500 | -0.3609 | No |
| 53 | GFPT1 |  |  | 4014 | -0.516 | -0.3648 | No |
| 54 | GALE |  |  | 4031 | -0.521 | -0.3609 | No |
| 55 | HAX1 |  |  | 4097 | -0.540 | -0.3661 | No |
| 56 | GOT1 |  |  | 4102 | -0.541 | -0.3597 | No |
| 57 | TPI1 |  |  | 4134 | -0.550 | -0.3582 | No |
| 58 | CLN6 |  |  | 4214 | -0.570 | -0.3658 | No |
| 59 | HDLBP |  |  | 4260 | -0.585 | -0.3666 | No |
| 60 | CITED2 |  |  | 4303 | -0.598 | -0.3667 | No |
| 61 | PYGL |  |  | 4349 | -0.614 | -0.3671 | Yes |
| 62 | GLRX |  |  | 4374 | -0.626 | -0.3633 | Yes |
| 63 | ERO1A |  |  | 4434 | -0.647 | -0.3660 | Yes |
| 64 | VCAN |  |  | 4464 | -0.661 | -0.3627 | Yes |
| 65 | SRD5A3 |  |  | 4502 | -0.682 | -0.3607 | Yes |
| 66 | NDST3 |  |  | 4519 | -0.689 | -0.3545 | Yes |
| 67 | QSOX1 |  |  | 4559 | -0.712 | -0.3525 | Yes |
| 68 | LDHA |  |  | 4574 | -0.722 | -0.3455 | Yes |
| 69 | ADORA2B |  |  | 4590 | -0.729 | -0.3386 | Yes |
| 70 | UGP2 |  |  | 4595 | -0.732 | -0.3295 | Yes |
| 71 | NT5E |  |  | 4607 | -0.737 | -0.3218 | Yes |
| 72 | SDC1 |  |  | 4663 | -0.770 | -0.3220 | Yes |
| 73 | CENPA |  |  | 4683 | -0.788 | -0.3151 | Yes |
| 74 | PLOD1 |  |  | 4685 | -0.788 | -0.3047 | Yes |
| 75 | MIF |  |  | 4692 | -0.790 | -0.2953 | Yes |
| 76 | G6PD |  |  | 4698 | -0.796 | -0.2855 | Yes |
| 77 | AURKA |  |  | 4906 | -0.973 | -0.3123 | Yes |
| 78 | KDELR3 |  |  | 4912 | -0.981 | -0.3001 | Yes |
| 79 | SLC16A3 |  |  | 4918 | -0.983 | -0.2879 | Yes |
| 80 | ME1 |  |  | 4950 | -1.019 | -0.2802 | Yes |
| 81 | PKM |  |  | 4986 | -1.071 | -0.2726 | Yes |
| 82 | HMMR |  |  | 4993 | -1.080 | -0.2592 | Yes |
| 83 | PFKP |  |  | 5042 | -1.173 | -0.2527 | Yes |
| 84 | DEPDC1 |  |  | 5053 | -1.193 | -0.2387 | Yes |
| 85 | TXN |  |  | 5080 | -1.246 | -0.2269 | Yes |
| 86 | TGFBI |  |  | 5104 | -1.305 | -0.2139 | Yes |
| 87 | P4HA2 |  |  | 5127 | -1.346 | -0.2000 | Yes |
| 88 | ENO1 |  |  | 5146 | -1.410 | -0.1846 | Yes |
| 89 | MET |  |  | 5153 | -1.438 | -0.1665 | Yes |
| 90 | CDK1 |  |  | 5164 | -1.512 | -0.1481 | Yes |
| 91 | PYGB |  |  | 5175 | -1.542 | -0.1293 | Yes |
| 92 | TPBG |  |  | 5221 | -1.800 | -0.1138 | Yes |
| 93 | EGFR |  |  | 5233 | -1.904 | -0.0904 | Yes |
| 94 | DCN |  |  | 5237 | -1.928 | -0.0651 | Yes |
| 95 | COL5A1 |  |  | 5276 | -2.605 | -0.0375 | Yes |
| 96 | TFF3 |  |  | 5283 | -2.980 | 0.0013 | Yes |
Table: GSEA details [plain text format]

  

Fig 2: HALLMARK\_GLYCOLYSIS: Random ES distribution      
 Gene set null distribution of ES for **HALLMARK\_GLYCOLYSIS**
